# Water Use Efficiency and Physiological Response of Rice Cultivars under Alternate Wetting and Drying Conditions

**DOI:** 10.1100/2012/287907

**Published:** 2012-12-18

**Authors:** Yunbo Zhang, Qiyuan Tang, Shaobing Peng, Danying Xing, Jianquan Qin, Rebecca C. Laza, Bermenito R. Punzalan

**Affiliations:** ^1^Crop Physiology, Ecology, and Production Center (CPEP), Hunan Agricultural University, Changsha, Hunan 410128, China; ^2^Agricultural College, Yangtze University, Jingzhou, Hubei 434025, China; ^3^Crop Physiology and Production Center (CPPC), MOA Key Laboratory of Crop Physiology, Ecology and Cultivation (The Middle Reaches of Yangtze River), Huazhong Agricultural University, Wuhan, Hubei 430070, China; ^4^Crop and Environmental Sciences Division, International Rice Research Institute (IRRI), DAPO Box 7777, Metro Manila, Philippines

## Abstract

One of the technology options that can help farmers cope with water scarcity at the field level is alternate wetting and drying (AWD). Limited information is available on the varietal responses to nitrogen, AWD, and their interactions. Field experiments were conducted at the International Rice Research Institute (IRRI) farm in 2009 dry season (DS), 2009 wet season (WS), and 2010 DS to determine genotypic responses and water use efficiency of rice under two N rates and two water management treatments. Grain yield was not significantly different between AWD and continuous flooding (CF) across the three seasons. Interactive effects among variety, water management, and N rate were not significant. The high yield was attributed to the significantly higher grain weight, which in turn was due to slower grain filling and high leaf N at the later stage of grain filling of CF. AWD treatments accelerated the grain filling rate, shortened grain filling period, and enhanced whole plant senescence. Under normal dry-season conditions, such as 2010 DS, AWD reduced water input by 24.5% than CF; however, it decreased grain yield by 6.9% due to accelerated leaf senescence. The study indicates that proper water management greatly contributes to grain yield in the late stage of grain filling, and it is critical for safe AWD technology.

## 1. Introduction

Rice (*Oryza sativa* L.) is a major staple food for the world's population with about two-thirds of the total rice production grown under irrigation [1]. In the past 10 years, the growth of rice yield has dropped below 1% per year worldwide, but an increase of more than 1.2% per year is required to meet the growing demand for food [2]. Rice production in Asia is increasingly constrained by water limitation [3] and increasing pressure to reduce water use in irrigated production as a consequence of global water crisis [4]. Guerra [5] reported that 60% of the world's irrigated fields are in Asia, half of which are devoted to rice production. Irrigated lowland rice consumes more than 50% of total freshwater, and irrigated flooded rice requires two or three times more water than other cereal crops, such as wheat and maize [6]. In addition, rice production is facing increasing competition with rapid urban and industrial development in terms of freshwater resource [7]. The need for “more rice with less water” is crucial for food security, and irrigation plays a greater role in meeting future food needs than it has in the past [8].

Continuous flooding (CF) provides a favorable water and nutrient supply under anaerobic conditions. However, the conventional system consumes a large amount of water [9]. A number of water-saving irrigation (WSI) technologies to reduce water use, to increase water use efficiency, and to maintain or increase production for rice-based systems have been developed [10, 11]. One of the most commonly practiced WSI techniques is alternate wetting and drying (AWD) irrigation [7, 12, 13]. In AWD, water is applied to irrigate the field depending on the weather condition or until some fine cracks appear on the soil surface.

Water use efficiency (WUE) is defined as the units of yield produced per unit of available water [14]. Crop WUE is especially an important consideration where available water resources are limited or diminishing. According to Fisher [15], WUE among plant varieties is essentially the same while gene improvement cannot change WUE [16]. However, WUE could be enhanced under restricted water by increasing transpiration and evaporation rate and by improving harvest index.

Nitrogen is one of the most important agricultural inputs to increase yield, and its use and uptake are affected by availability of water. Fertilizer application can improve both the crop yield and WUE. Hatfield [17] reported that the vital issue of nutrition is how to fertilize and improve WUE under restricted water conditions. Chlorophyll meter (SPAD) is a convenient tool to estimate leaf nitrogen concentration of rice plant. It is a simple, quick, and nondestructive method [18], and SPAD values are closely correlated with leaf N concentration [19]. Senescence is a genetically programmed process that involves remobilization of nutrients from vegetative tissues to grains [20, 21]. In China's super rice, too much use of nitrogen fertilizer leads to slow grain filling and low harvest index because leaves stay “green” for a too long time in the late stage of grain filling [22]. Water stress imposed during grain filling, especially at the early stage, usually results in a reduction in grain weight [23].

Water deficiency can accelerate plant senescence and lead to a faster and better remobilization of carbon from vegetative tissues to the grain [24]. Grain filling, which is an essential determinant of grain yield in cereal crops, is characterized by its duration and rate; these parameters correlated with other yield-related components of rice grain filling rate were more important than duration [25]. Grain filling rate was positively correlated with actual panicle weight and 100-grain weight and was negatively correlated with panicles m^−2^ [26].

In our current study, we compared hybrid rice varieties and inbred varieties under two N rates (low N, high N) and two water management treatments (AWD, CF). The objectives of this study were (1) to determine rice yield potential and water use efficiency under two N rates and two water management methods, (2) to identify the factors that contribute to increased yield and water productivity under these conditions, and (3) to determine if there exist interactions among N, water management, and varieties.

## 2. Materials and Methods

The field experiments were conducted for three consecutive seasons (2009 dry season (DS), 2009 wet season (WS), and 2010 DS) in the same field at the International Rice Research Institute (IRRI) farm, Los Baños (14°11′N, 121°15′E, and 21 m als), Philippines. The soil was an Aquandic Epiaquoll with pH 6.2; 20.0 g kg^−1^ organic C; 2.0 g kg^−1^ total N; 11.4 mg kg^−1^ Olsen P; 0.43 cmol kg^−1^ exchangeable K and 34.5 cmol kg^−1^ cation exchange capacity; and 58.3% clay, 34.0% silt, and 8.0% sand. The soil test was based on samples taken from the upper 20 cm of the soil before transplanting in 2010 DS.

The experimental design was split-split plot with four replications in the three seasons. The main plots were two water management treatments (AWD and CF). The subplots were two N treatments: low N rate (60 kg ha^−1^ in WS, 100 kg ha^−1^ in DS) and high N (120 kg ha^−1^ in WS, 200 kg ha^−1^ in DS). The sub-subplots were four rice varieties; they belong to two groups: hybrid rice (IR72 and PSBRc80) and inbred varieties (IR82372H and Mestizo7). SL8-H was replaced with Mestizo7 because of its disease susceptibility in the WS.

In the CF plots, ponded water was kept with a depth of 3–5 cm during the 7 days after transplanting until the 7 days before maturity. In the AWD plots, soil water potential was measured with two porous-cup tensiometers installed at 20 cm and 40 cm depth. The depth of groundwater table was monitored using piezometers in open-bottom PVC tubes installed at a depth of 100 cm. Holes were perforated on all sides of the tube. When the ponded water dropped to 15 cm below the soil surface, then irrigation was applied to reflood the field up to 5 cm in AWD treatment. This cycle was repeated throughout the season. The first AWD treatment was initiated in the 3 weeks after transplanting. The irrigated water of each plot was measured using a 90° boxed Weir connected to an irrigation outlet. Daily mean temperature and rainfall were recorded from the weather station adjacent to the experimental site. Total water input = the amount of irrigated water applied + rainfall. Water productivity = grain yield/total amount of water supplied.

Pregerminated seeds were sown in seedling trays to produce uniform seedings. Fourteen-day-old seedlings were manually transplanted on January 6, June 10, and January 14 for 2009 DS, 2009 WS, and 2010 DS, respectively. Four seedlings per hill were transplanted at a hill spacing of 20 cm × 20 cm. Insects, diseases, and weeds were intensively controlled by using approved pesticides to avoid biomass and yield loss. Fertilizers were manually broadcasted and incorporated during basal application: 30 kg P ha^−1^, 40 kg K ha^−1^, and 5 kg Zn ha^−1^in the DS and 15 kg P ha^−1^, 20 kg K ha^−1^, and 2.5 kg Zn ha^−1^ in the WS. Nitrogen in the form of urea was applied. During DS, low N rate was supplied with 40, 20, 40, and 20 kg N ha^−1^ at basal, midtillering, panicle initiation, and booting, respectively. High N rate corresponded to 60-40-60-40 kg N ha^−1^. During WS, low N rate was lowered to 20-10-20-10 kg N ha^−1^ while the rate for high N was reduced to 30-20-30-20 kg N ha^−1^. In the dry season, total N rate was 120 and 200 kg ha^−1^ for the low and high N rates, respectively. In the wet season experiments, total N rate was 60 and 100 kg ha^−1^ for the low and high N rates, respectively.

The soil water content (SWC) of the soil was monitored when water was deficient in the AWD treatment in 2010 DS. In each plot, soil samples were taken every 2 days using a core sampler. Fresh weight of the soil samples was measured immediately. Dry weight was obtained after oven drying at 105°C for 24 h. The soil water content was calculated following the equation: SWC = 100 × (fresh weight − dry weight)/fresh weight. Three varieties (IR72, IR82372H, and SL-8H) were used to measure grain filling and SPAD value. At the onset of flowering, 150 panicles headed on the same day were initially tagged from the high N plots. Among these panicles, ten were taken every two days from heading until maturity. The SPAD value of its flag leaf was also measured before sampling. Dry weights of the spikelets were determined after oven drying at 70°C to constant weight.

For growth analysis, 12 hills were sampled from each plot at flowering to measure plant height, stem number, leaf area index, and aboveground total dry weight. Plant height was measured from the plant base to the tip of the highest leaf. Plants were separated into green leaves and stems. Green leaf area was measured with a leaf area meter (LI-3000, LI-COR, Lincoln, NE, USA) and expressed as leaf area index. The dry weight of each component was determined after oven drying at 70°C to constant weight. Total dry weight was the sum of the weights of green leaves and stems. At maturity, 12 hills were taken diagonally from a 5 m^2^ area in each plot where grain yield was determined to measure the above ground total dry weight, harvest index, and yield components. Panicles of each hill were counted to determine the panicle number per m^2^. Plants were separated into straw and panicles. Straw dry weight was determined after oven drying at 70°C to constant weight. Panicles of all 12 hills were hand threshed and filled spikelets were separated from unfilled spikelets by submerging them in tap water. Three subsamples each of 30 g filled spikelets and 2 g unfilled spikelets were taken to determine the number of spikelets. Dry weights of rachis and filled and unfilled spikelets were measured after oven drying at 70°C to constant weight. Aboveground total dry weight was the total dry matter of straw, rachis, and filled and unfilled spikelets. Spikelets per panicle, grain filling percentage (100 × filled spikelet number/total spikelet number), and harvest index (100 × filled spikelet weight/aboveground total dry weight) were calculated. Grain yield was determined from a 5 m^2^ area in each plot and adjusted to the standard moisture content of 0.14 g H_2_O g^−1^ fresh weight. Grain moisture content was measured with a digital moisture tester (DMC-700, Seedburo, Chicago, IL, USA).

Data were analyzed following the analysis of variance (SAS Institute) and means were compared based on the least significant difference test (LSD) at the 0.05 probability level [27].

## 3. Results and Discussion

**Figure 1:**
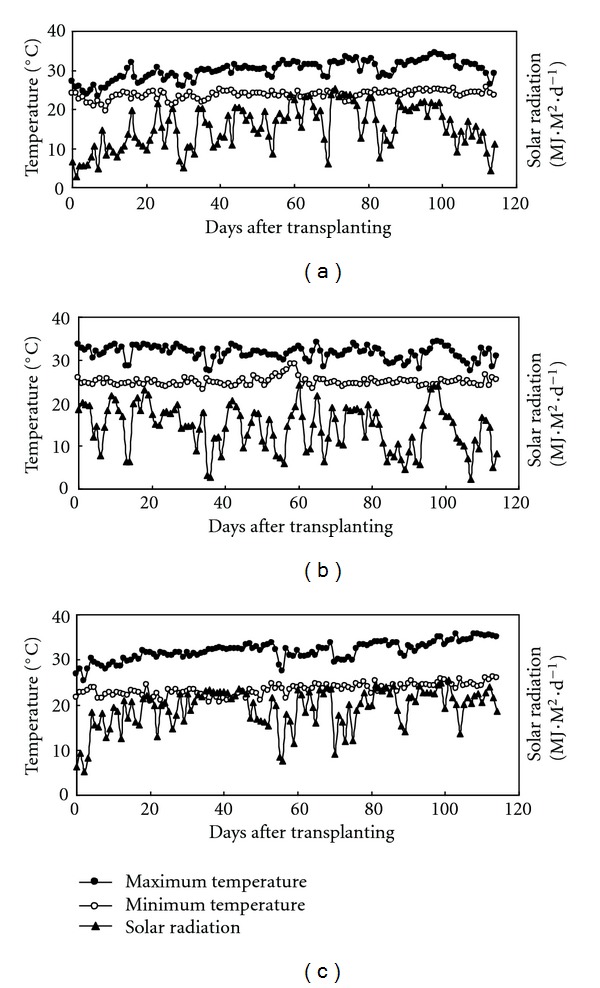
Daily maximum and minimum temperatures and solar radiation during rice-growing seasons at the IRRI farm in 2009 DS (a), 2009 WS (b), and 2010 DS (c).

Average temperatures during the growing season in 2009 DS were 1.1–1.3°C higher than that in the 2009 WS (Figure 1). Seasonal mean values of maximum temperature were 29.9°C in 2009 DS, 31.2°C in 2009 WS, and 31.9°C in 2010 DS, whereas seasonal mean minimum temperatures were 23.6, 24.7, and 23.3°C for 2009 DS, 2009 WS, and 2010 DS, respectively. Higher daily minimum temperature and lower radiation were observed in the WS compared with the DS. No significant differences in daily maximum temperature between the two DS were observed. Seasonal mean radiation was 15.3, 13.9, and 19.3 MJ M^−2^ day^−1^ in 2009 DS, 2009 WS, and 2010 DS, respectively. The difference in radiation during the growing season between the DS and WS in 2009 was about 15% and about 10% between the two DS.

Total rainfall of each season was 349, 1079, and 92 mm in 2009 DS, 2009 WS, and 2010 DS, respectively (Table 1). There was about 67% difference in rainfall during the growing season between the DS and WS in 2009 and about 73% difference between the two DS. The total amount of water input (irrigation plus rainfall) in the AWD was 876, 1184, and 833 mm in 2009 DS, 2009 WS, and 2010 DS, which was 7.2%, 5.3%, and 24.5% less than the CF, respectively. CF greatly consumed more water than AWD, especially in the 2010 DS.

**Figure 2:**
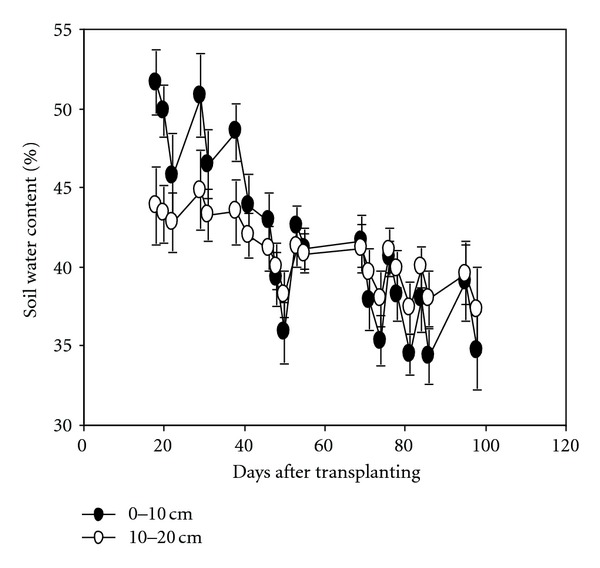
Change in soil water content during the growing season under AWD in 2010 DS at the IRRI farm. Data were the means across two rates and four varieties; N rate and variety had no significant effect on soil water content.

Soil water content during the growing period under AWD in 2010 DS was shown in Figure 2. Analysis showed that N rate and variety had no significant effect on soil water content; the soil water content at 0–10 cm depth was higher than that of 10–20 cm during the vegetative stage. However, it was lower during the late growth stage because several reirrigations will influence soil structure. In many previous studies [7, 28], the time of irrigation was determined by soil water potential, and 0–20 kpa in the root zone was defined as mild stress and 50–80 Kpa as severe stress. This study followed an irrigation scheme according to soil water content of the upper 20 cm soil in 2010 DS and took SWC of 40% and 30% as irrigation threshold at PI and grain filling stage, respectively. Compared with soil water potential, it is a more accurate and simpler method to measure soil water content in field.

Interaction effects of variety, water management, and N rate in all the three experiments were not significant. Grain yield was not significantly different between AWD and CF across the three seasons (Table 2). Varietal differences in grain yield were significant in the two DS experiments, but not significant in the 2009 WS (Table 3). Average yield of AWD was 7.22 t ha^−1^ in 2009 DS, 5.07 t ha^−1^ in 2009 WS, and 8.01 t ha^−1^ in 2010 DS, respectively. Compared with CF, AWD reduced water input of 7.2%, 5.3%, and 24.5% and lost grain yield of 5.3%, 2.9%, and 6.9% in 2009 DS, 2009 WS, and 2010 DS, respectively. Cabangon [28] reported that mild stress AWD reduced irrigation water input by 8%–20% and severe stress by 19%–25% compared with CF. In this study, there was a large amount of rainfall in both 2009 DS and 2009 WS, which resulted in high water input particularly in 2009 WS. Earlier studies showed that even a 2%–70% water irrigation reduction would not lead to rice yield decrease [5, 7]. In this study, grain yield was not significantly different between AWD and CF in all the three experiments. CF produced a greater yield due to its higher grain weight.

Nitrogen rate had a significant effect on grain yield in all the three experiments. In this study a significantdifference in water productivity between N treatments onlyin the normal dry season such as 2010 DSwas observed. In the 2010 DS, CF received 16 irrigations from transplanting to maturity, while 10 irrigations were applied to AWD. The number of irrigation was reduced in 2009 WS, when 2 and 1 irrigations were applied to CF and AWD, respectively. The differences in water productivity between AWD and CF treatments were insignificant. Water productivity in the two DS ranged from 0.78 to 1.09, which was 2.0–2.4 times higher than in 2009 WS. No significant interactions were observed in terms of variety, water management and, N rate. Varieties with higher yield had greater WUE. AWD received higher WUE than CF due to the decrease in water input. Using high nitrogen fertilization and high yield varieties were the two ways to improved water productivity in this study, as discussed by Hatfield [17].

The difference in grain yield between the hybrid and inbred varieties was relatively slight, except in 2010 DS. Nitrogen rate had a significant effect on grain yield in all the three experiments. Significant differences in grain weight between the AWD and CF treatments were observed in 2010 DS. Panicles per m^2^ and spikelets per m^2^ were significantly higher in high N than low N. Among the four varieties, the hybrid ones had more spikelets number per m^2^ compared with inbreds ones (Table 4). Hybrids had an average of 109 spikelets per panicle, which was 23% higher than the inbreds. IR72 had the highest panicles per m^2^ among the varieties. In general, grain filling percentage was the lowest in IR82372H and the highest in IR72. Grain weight of the hybrid variety SL-8H was more than 26.0 mg in two DS. Spikelet number per m^2^ was higher in the DS than in the WS and higher in 2010 DS than in 2009 DS.

The LAI at flowering was significantly higher in high N than low N in the two DS. LAI in hybrids was higher than in inbreds in 2009 WS and 2009 DS (Table 5). Differences in the total dry weight at maturity were significant in N treatments, but not significant in water treatments across the three seasons. Harvest index (HI) was significantly higher in hybrids than the inbreds in 2009 WS and 2009 DS. Both HI and LAI (leaf area index) at flowering were higher in the DS than in the WS. Average HI was 49.9% in 2009 DS and 44.8% in 2010 DS.

**Figure 3:**
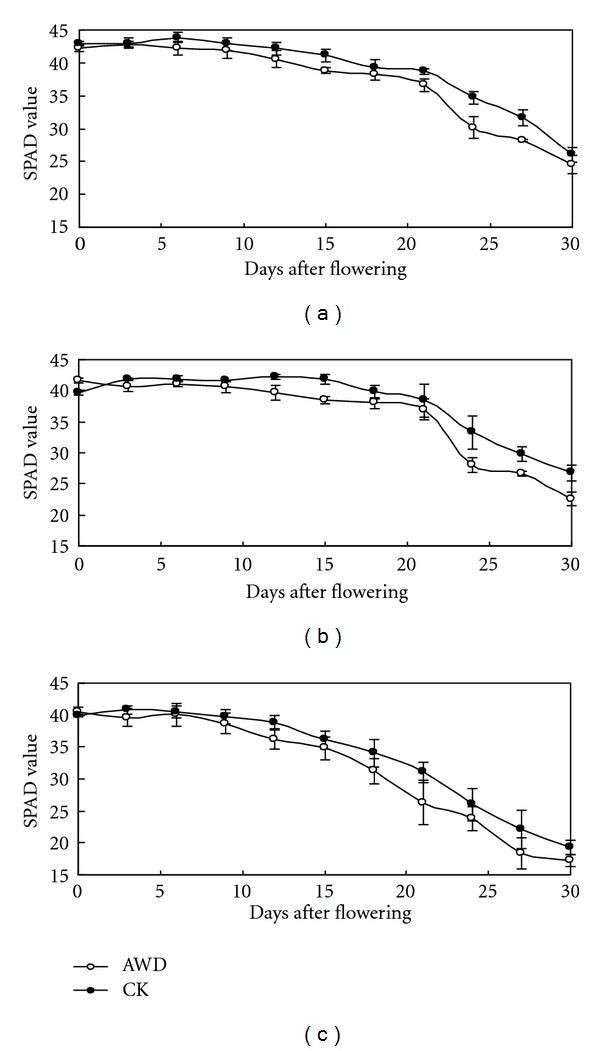
SPAD values after flowering under AWD and CF in 2010 DS at the IRRI farm. Three varieties IR72 (a), IR82372H (b), and SL-8H (c) were used in the experiment at high N level.

SPAD values were significantly different between AWD and CF in three varieties at the grain filling stage in 2010 DS (Figure 3). The SPAD values of AWD at the first flowering were slightly higher than those of CF. SPAD values rapidly decreased on the 21st day after flowering in IR72 and IR82372H, while values decreased on the 15th day after flowering in SL-8H due to leaf senescence. However, on the 30th day after flowering the SPAD values were obviously lower than those of the CF. Among the three varieties, SPAD values of IR72 and IR8237H were significantly higher than those of SL-8H, particularly on the 30th day after flowering. Therefore, the SPAD value of AWD was higher in the early grain filling stage and lower in the later stage, but CF kept stable leaf senescence and high SPAD value in the later grain filling stage.

**Figure 4:**
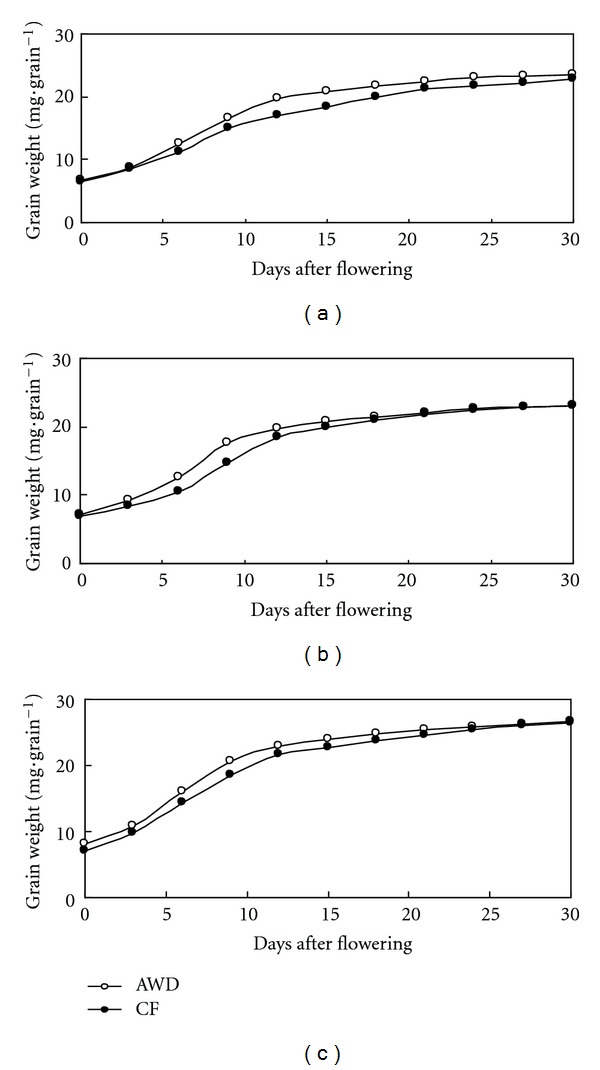
Grain weight after flowering under AWD and CF in 2010 DS. Three varieties IR72 (a), IR82372H (b), and SL-8H (c) were used in the experiment at high N level.

**Figure 5:**
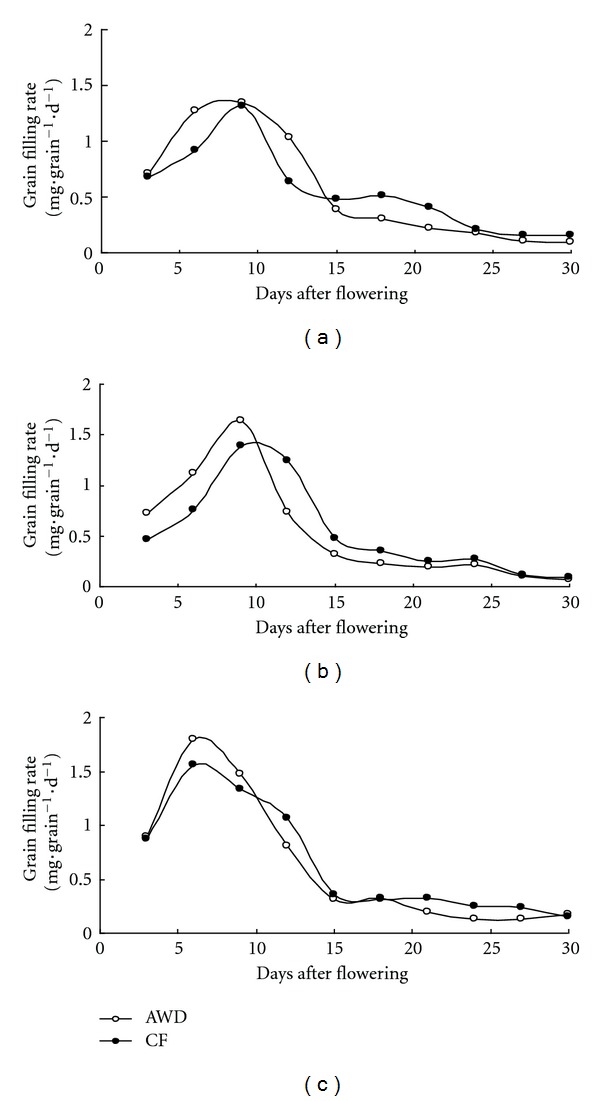
Grain filling rate after flowering under AWD and CF in 2010 DS. Three varieties IR72 (a), IR82372H (b), and SL-8H (c) were used in the experiment at high N level.

Grain filling rates (mg · grain^−1^ · day^−1^) were significantly different between AWD and CF in all the three varieties in grain filling stage (Figures 4 and 5). Grain filling rate of AWD was high at the early grain filling stage and low at the late grain filling stage, but under CF condition grain filling rate was still high at the late grain filling stage. The maximum grain filling rate occurred on the 9th day after flowering in both IR72 and IR8237H and on the 6th day in SL-8H. The maximum grain filling values between AWD and CF were 1.35 mg · grain^−1^ · day^−1^ and 1.31 mg · grain^−1^ · day^−1^ for IR72, 1.64 mg · grain^−1^ · day^−1^ and 1.39 mg · grain^−1^ · day^−1^ for IR82372H, and 1.80 mg · grain^−1^ · day^−1^ and 1.56 mg · grain^−1^ · day^−1^ for SL-8H, respectively. The average grain filling rate was 0.77 mg · grain^−1^ · day^−1^ in IR72, 0.77 mg · grain^−1^ · day^−1^ in IR82372H, and 0.89 mg · grain^−1^ · day^−1^ in SL-8H. Cereal grains accumulate carbohydrates, proteins, and fatty acids via different pathways during their development [29]. Grain filling plays an important role in grain weight, which is an essential determinant of grain yield in cereal crops, and is characterized by its duration and rate [25]. AWD treatment increased the grain filling rate and shortened grain filling period. Active grain filling period was shortened by 2.1 days and grain filling rate increased by 0.15 mg per day per grain compared with CF. The SPAD value of AWD was higher in the early grain filling stage and lower in the later stage, but CF kept stable leaf senescence and high SPAD value in the later grain filling stage. AWD reduced water input by 25% in tropical area in 2010 DS but decreased grain yield by 5% due to accelerated leaf senescence. High SPAD value and grain filling rate in the later grain filling stage were partially responsible for the high yield of flooded water management. Water deficiency lead to hormonal change, which enhanced whole plant senescence and accelerated grain filling [22].

In conclusion, interaction effects among variety, water management, and N rate were not significant under tropical condition. Grain yield was not significantly different between AWD and CF in all the seasons through saving water input. Using high nitrogen fertilization and high yield varieties were the two ways to improve water productivity in this study; severe water stress during late grain filling stage accelerated grain filling rate, shortened the grain filling period, and enhanced whole plant senescence, thus reducing grain weight. The study indicated that proper water management greatly contributed to grain yield in the late grain filling stage, and it was critical for safe AWD technology.

## Figures and Tables

**Table 1:** Rainfall and total water supply (irrigation plus rainfall) of the four rice varieties grown under two water management treatments and two N rates at IRRI farm in the three consecutive seasons.

N	Irrigation (mm)		Rainfall	Total water input (mm)		Reduction
	AWD	CF			AWD	CF
2009 DS						
LN	517	606	349	866	955	9.3
HN	537	584	349	886	933	5.0
2009 WS						
LN	107	168	1079	1186	1247	4.9
HN	102	172	1079	1181	1251	5.6
2010 DS						
LN	753	1024	92	845	1116	24.3
HN	729	998	92	821	1090	24.7

Data are the means across four varieties. Variety had insignificant effect on the amount of water supply.

**Table 2:** Analysis of variance for grain yield and water use efficiency (WUE) in the three consecutive seasons at IRRI farm, Philippines.

Year	2009 DS		2009 WS		2010 DS	
Source of variation	Yield	WUE	Yield	WUE	Yield	WUE
Water regime (W)	ns	ns	ns	ns	ns	∗
Nitrogen (N)	∗	ns	∗∗	∗	∗∗	∗
Variety (V)	∗	∗	∗	ns	∗	∗
W × N	ns	ns	ns	ns	ns	ns
W × V	ns	ns	ns	ns	ns	ns
N × V	ns	ns	ns	ns	ns	ns
W × N × V	ns	ns	ns	ns	ns	ns

*Significance at the 0.05 level based on analysis of variance.

**Significance at the 0.01 level based on analysis of variance.

ns: denotes nonsignificance based on analysis of variance.

**Table 3:** Grain yield and water productivity of the four rice varieties grown under two water management treatments and two N rates at the IRRI farm for the three consecutive seasons.

Variety	Grain yield (t ha^−1^)				Water productivity (kg m^−3^)			
	LN		HN		LN		HN	
	AWD	CF	AWD	CF	AWD	CF	AWD	CF
2009 DS								
IR72	7.45^a^	7.80^b^	7.74^ab^	7.75^b^	0.86^a^	0.82^ab^	0.87^ab^	0.83^b^
PSBRc80	7.52^a^	7.45^b^	7.67^ab^	8.29^ab^	0.87^a^	0.78^b^	0.87^ab^	0.89^a^
IR82372H	7.66^a^	8.16^ab^	8.55^a^	9.09^a^	0.88^a^	0.85^a^	0.97^a^	0.97^a^
SL-8H	7.90^a^	8.23^a^	7.30^b^	8.48^ab^	0.91^a^	0.86^a^	0.82^b^	0.91^a^

Mean	7.63	7.91	7.82	8.40	0.88	0.83	0.88	0.90

2009 WS								
IR72	4.96^a^	5.05^a^	5.6^a^	5.63^a^	0.42^a^	0.40^a^	0.47^a^	0.45^a^
PSBRc80	5.11^a^	4.92^a^	5.03^a^	5.29^a^	0.43^a^	0.39^a^	0.43^a^	0.42^a^
IR82372H	4.73^a^	4.97^a^	5.30^a^	5.47^a^	0.40^a^	0.40^a^	0.45^a^	0.44^a^
Mestizo7	4.64^a^	5.12^a^	5.22^a^	5.34^a^	0.39^a^	0.41^a^	0.44^a^	0.43^a^

Mean	4.86	5.02	5.29	5.43	0.41	0.4	0.45	0.44

2010 DS								
IR72	7.51^a^	7.69^b^	8.93^a^	8.74^b^	0.89^a^	0.69^b^	1.09^a^	0.80^b^
PSBRc80	7.57^a^	7.94^b^	8.86^a^	9.50^a^	0.90^a^	0.71^b^	1.08^a^	0.87^a^
IR82372H	7.21^a^	7.97^b^	8.35^b^	8.74^b^	0.85^a^	0.71^b^	1.02^a^	0.80^b^
SL-8H	7.53^a^	8.92^a^	8.14^b^	9.37^a^	0.89^a^	0.80^a^	0.99^a^	0.86^a^

Mean	7.46	8.13	8.57	9.09	0.88	0.73	1.05	0.86

Data are the means across two N rates. Within a column for each season, means followed by the same letters are not significantly different according to LSD (0.05).

**Table 4:** Yield components of the four rice varieties grown under two water management treatments and two N rates at the IRRI farm for the three consecutive seasons.

	Spikelets panicle^−1^		Panicles m^2^		Grain filling (%)		Grain weight (mg)	
	AWD	CF	AWD	CF	AWD	CF	AWD	CF
2009 DS								
IR72	80.2^c^	83.4^c^	412.3^a^	441.2^a^	87.9^a^	85.5^a^	23.2^d^	23.0^d^
PSBRc80	101.7^b^	104.6^b^	370.3^b^	363.8^b^	82.8^ab^	81.6^b^	23.9^c^	23.6^c^
IR82372H	120.0^a^	117.4^a^	330.2^c^	344.8^b^	78.3^b^	77.1^c^	24.4^b^	24.5^b^
SL-8H	119.8^a^	124.8^a^	228.3^d^	301.1^c^	81.3^b^	83.0^ab^	26.8^a^	27.1^a^

Mean	105.4	107.6	335.3	362.7	82.6	81.8	24.6	24.6

2009 WS								
IR72	79.6^b^	81.0^b^	351.1^a^	351.6^a^	74.0^a^	75.9^ab^	21.9^c^	22.1^c^
PSBRc80	94.3^a^	96.9^a^	295.6^b^	296.1^b^	74.9^a^	76.7^a^	22.7^b^	22.5^b^
IR82372H	98.5^a^	99.6^a^	307.8^b^	298.7^b^	66.6^b^	71.5^b^	23.2^a^	23.4^a^
Mestizo7	100.4^a^	95.2^a^	291.2^b^	298.2^b^	72.1^a^	74.7^ab^	23.4^a^	23.5^a^

Mean	93.2	93.2	311.4	311.2	71.9	74.7	22.8	22.9

2010 DS								
IR72	70.5^b^	72.9^c^	517.5^a^	504.5^a^	90.0^a^	89.8^a^	22.4^d^	22.7^d^
PSBRc80	97.4^a^	98.8^b^	424.0^b^	420.1^b^	81.9^bc^	83.6^ab^	23.0^c^	23.1^c^
IR82372H	108.9^a^	106.6^ab^	390.3^c^	407.0^b^	79.0^c^	82.4^b^	23.5^b^	23.8^b^
SL-8H	105.9^a^	110.1^a^	346.9^d^	344.0^c^	84.8^b^	85.9^ab^	26.2^a^	26.5^a^

Mean	95.7	97.1	419.7	418.9	83.9	85.4	23.8	24.0

Data are the means across two N rates. Within a column for each site, means followed by the same letters are not significantly different according to LSD (0.05).

**Table 5:** Growth duration, leaf area index (LAI) at flowering, harvest index, and total dry weight of the four rice varieties grown under two water management treatments and two N rates at the IRRI farm for the three consecutive seasons.

	Growth duration (days)		LAI at flowering		Total dry weight (g m^−2^)		Harvest index (%)	
	AWD	CF	AWD	CF	AWD	CF	AWD	CF
2009 DS								
IR72	104	104	5.49^b^	5.35^c^	1481^a^	1557^a^	45.5^b^	46.4^c^
PSBRc80	106	104	5.56^b^	5.58^bc^	1481^a^	1459^b^	50.1^a^	50.1^b^
IR82372H	100	100	6.63^a^	6.26^ab^	1441^b^	1464^b^	50.6^a^	52.2^a^
SL-8H	106	106	6.82^a^	6.71^a^	1478^a^	1609^a^	52.1^a^	52.4^a^

Mean	104	104	6.13	5.98	1470	1522	49.6	50.3

2009 WS								
IR72	101	102	3.16^b^	3.31^a^	1089^a^	1117^a^	41.7^b^	42.7^c^
PSBRc80	103	103	3.30^ab^	3.53^a^	1078^a^	1119^a^	43.8^b^	44.3^bc^
IR82372H	102	102	3.56^a^	3.50^a^	1078^a^	1105^a^	43.5^b^	44.9^b^
Mestizo7	100	100	3.55^a^	3.71^a^	1045^b^	1055^a^	47.2^a^	47.1^a^

Mean	102	102	3.39	3.51	1073	1099	44.1	44.8

2010 DS								
IR72	105	106	4.70^a^	5.15^a^	1545^b^	1560^a^	47.7^b^	48.1^b^
PSBRc80	107	107	4.63^a^	5.40^a^	1594^b^	1578^a^	48.6^ab^	50.7^ab^
IR82372H	100	100	4.72^a^	5.05^a^	1554^b^	1631^a^	50.4^a^	50.0^a^
SL-8H	111	111	5.04^a^	5.01^a^	1658^a^	1667^a^	49.1^ab^	51.7^ab^

Mean	106	106	4.77	5.15	1588	1609	49.0	50.1

Data are the means across two N rates. Within a column for each season, means followed by the same letters are not significantly different according to LSD (0.05).
